# Infection with a novel polymycovirus enhances growth, conidiation and sensitivity to UV-B irradiation of the entomopathogenic fungus *Metarhizium anisopliae*

**DOI:** 10.3389/fmicb.2023.1214133

**Published:** 2023-07-04

**Authors:** Ping Wang, Guogen Yang, Hanwen Lu, Bo Huang

**Affiliations:** ^1^Anhui Provincial Key Laboratory of Microbial Pest Control, Anhui Agricultural University, Hefei, China; ^2^School of Plant Protection, Anhui Agricultural University, Hefei, China

**Keywords:** Metarhizium anisopliae, polymycovirus, growth, conidiation, UV-B irradiation

## Abstract

*Metarhizium anisopliae* is a well-studied entomopathogenic fungus that is widely used in biological control programs. The presence of polymycoviruses in this fungus is common, but their effects on fungal development and stress tolerance are not well understood. In this study, we report the discovery of a novel double-stranded RNA virus, named Metarhizium anisopliae polymycovirus 1 (MaPmV1), which comprises four dsRNAs ranging from 2.4 to 1.4 kbp in length. Phylogenetic analysis revealed that MaPmV1 belongs to the *Polymycoviridae* family. Biological comparison between MaPmV1-infected (Vi) and -free (Vf) isogenic lines showed that MaPmV1 remarkably enhances the growth rate and conidiation of the host fungus. The upregulation of growth- and conidiation-related genes in Vi strains supports this finding. In addition, MaPmV1 increases the sensitivity of the host to UV-B irradiation, which is evidenced by the downregulation of DNA damage repair genes in Vi strains. However, MaPmV1 does not appear to have any significant impact on the virulence of *M. anisopliae*. Furthermore, overexpression of individual viral proteins in *M. anisopliae* did not result in any significant phenotypic alterations, indicating that MaPmV1-mediated changes are not related to a single viral protein. Overall, our findings suggest that mycoviruses can be exploited to enhance fungal development in entomopathogenic fungi, which may lead to improved conidium production on a large scale.

## Introduction

*Metarhizium* spp., an important group of entomopathogenic fungi, plays a crucial role in regulating insect populations in nature. Among them, *M. anisopliae* is the most well-studied and widely used in biological control programs due to its strong pathogenicity and broad host-specificity (Wang and Wang, 2017; Omuse et al., 2022). In Brazil, *M. anisopliae* has been successfully mass-produced and applied on a large scale to control sugarcane spittlebugs, making it one of the most effective fungal insecticides in the world (Iwanicki et al., 2019). In China, *M. anisoplia*e CQMa421 has been found to be as effective chemical pesticides in controlling major rice pests, and is applicable to both small- and industrial-scale agriculture (Peng et al., 2021).

For entomopathogenic fungi, conidia are essential structures responsible for dispersal and environmental resistance, as well as for the asexual propagation of fungal pathogens and host infection. In particular, entomopathogenic fungal conidia are commonly used as inoculum in biological control. However, the broad application of *M. anisopliae* is limited by its low conidiation rate, sensitivity to high temperature and ultraviolet stress, and slow insecticidal speed (Fang et al., 2012; Muniz-Paredes et al., 2017). Therefore, improving the spore production, stress resistance, and pathogenicity of the fungi is essential for expanding their use in biological control.

Mycoviruses are ubiquitous among fungi, including entomopathogenic fungi *Metarhizium*, and are parasitic in nature (Kotta-Loizou, 2021). Most mycoviruses are known to have double-stranded RNA genomes and belong to families such as *Amalgaviridae*, *Chrysoviridae*, *Megabirnaviridae*, *Partitiviridae*, *Polymycoviridae*, *Quadriviridae*, *Reoviridae*, *Totiviridae*, and the genus *Botybirnavirus*, as classified by the International Committee on Taxonomy of Viruses (ICTV). Recently, the family *Polymycoviridae* has been established, which includes the genus *Polymycovirus*. The members of this family have four to eight dsRNA segments consisting of non-conventionally encapsidated dsRNA genomes (Kotta-Loizou et al., 2022). *Polymycovirus* has been found to be infectious not only as a purified entity, but also as naked dsRNA (Kanhayuwa et al., 2015; Jia et al., 2017; Niu et al., 2018). While most mycoviruses are known to be latent infections, some polymycoviruses can alter host morphology, pigmentation, growth, sporulation, and virulence (Kanhayuwa et al., 2015; Jia et al., 2017; Kotta-Loizou and Coutts, 2017; Filippou et al., 2021).

*Metarhizium*, like many other fungi, is often associated with the presence of virus. Four dsRNA viruses have been identified and classified from *M. brunneum*, including two partitiviruses (MbPV1 and MbPV2) (Wang et al., 2020, 2021), an unassigned mycovirus (MbBV1) (Wang et al., 2022), and a polymycovirus (MbPmV1) (He et al., 2023). The effects of mycovirus infection on insect pathogenic fungi have been extensively studied, with mycovirus infection causing a significant decrease in host mycelial growth, conidiation, and virulence in *M. anisopliae* (Melzer and Bidochka, 1998; Gimenez-Pecci et al., 2002). Similarly, gammapartitivirus infection in the host *Cordyceps chanhua* can result in changes in fungal development and multi-stress tolerance (Zhu et al., 2022). However, the effects of polymycovirus infection on fungal development and stress tolerance in *Metarhizium* remain unclear.

Approximately 140 strains of *Metarhizium* spp. were collected from the Research Center for Entomogenous Fungi of Anhui Agricultural University (RCEF) and screened for the presence of dsRNA viruses using phenol/chloroform extraction and cellulose isolation methods. As a result, it was discovered that the *M. anisopliae* isolate RCEF3284 contained four dsRNA segments. Subsequently, we conducted further analysis to identify and characterize a novel mycovirus within this entomopathogenic fungus, which we named Metarhizium anisopliae polymycovirus 1 (MaPmV1). Through phylogenetic analysis and RdRp sequence comparisons, we placed MaPmV1 as a member of the *Polymycoviridae* family. Furthermore, we compared MaPmV1-infected (Vi) and virus-free (Vf) isogenic *M. anisopliae* isolates and found that MaPmV1 infection increased the growth rate, sporulation, and sensitivity to UV-B irradiation of the host. Notably, our results suggest that this mycovirus can enhance fungal development in entomopathogenic fungi and thus can be exploited to improve conidia production on a large scale.

## Materials and methods

### Fungal strains and culture conditions

*M. anisopliae* strain RCEF3284 was obtained from *Brontispa longissima* in Hainan Province, China in 2006. All *M. anisopliae* isolates were cultured on SDAY medium (1% w/v peptone, 4% w/v dextrose, 1% w/v yeast, 2% w/v agar) overlaid with a sterilized cellophane membrane to harvest mycelia. Conidia were collected after incubating the mycelia on potato dextrose agar (PDA, 20% w/v potato, 2% w/v glucose, and 2% w/v agar) at 25°C in the dark for 10 days.

### dsRNA extraction and purification

Viral dsRNA was extracted from fresh mycelia using *CF*-11 cellulose column chromatography, following the previously described protocol (Herrero et al., 2012). To remove residual DNA and ssRNA contaminants, dsRNA was treated with RNase-free DNase I and S1 nuclease (TaKaRa, Dalian, China). The resulting purified dsRNA preparations were then electrophoresed on 1.5% agarose gels, stained with ethidium bromide (0.1 mg/mL), and viewed on a UV transilluminator.

### Sequencing and analysis

The dsRNA sample was sequenced using an Illumina HiSeq 2,500 platform at BGI (Shenzhen, China). The total clean reads were assembled using Trinity (v2.1.1) for subsequent analysis. The assembled contigs were queried against the non-redundant protein database of the National Center for Biotechnology Information (NCBI) using BLASTx. The terminal sequences were established using RNA-ligase-mediated rapid amplification of cDNA ends (RLM-RACE), following a previously reported method (Coutts and Livieratos, 2003). To ensure accuracy, all RT-PCR products were cloned into the pMD18-T vector (TaKaRa, Dalian, China) and sequenced at least three times. The open reading frames (ORFs) were predicted using the NCBI ORFfinder.^1^ Homologous amino acid sequences were identified using the BLASTp program, and multiple sequence alignment was performed using the MAFFT program (Katoh et al., 2019). A maximum-likelihood (ML) phylogenetic tree was constructed based on amino acid alignments using the LG + G + I + F model in MEGA-X, with 1,000 bootstrap replicates (Kumar et al., 2018).

### Curing of virus from strain RCEF3284

To obtain a virus-free isogenic *M. anisopliae* isolate, single spore isolation was performed. A conidial suspension was obtained by vortex-mixing in 0.05% (v/v) Tween-80, filtered through sterile nonwoven fabric, and its concentration was measured using a hemocytometer. The suspension was then diluted to a concentration of 2 × 10^2^ conidia/mL and coated on PDA plates. The plates were incubated at 25°C to culture single-conidium isolates (Shafik et al., 2021). All single spore strains of RCEF3284 were screened for the presence of MaPmV1 by extracting dsRNA and verified by RT-PCR amplification.

### Plasmid construction and fungal transformation

To express MaPmV1-encoded ORF1-4 in virus-free strains, we used the vector pDHt-SK-bar-PgpdA, which contains the ammonium glufosinate resistance gene (*bar*), the gpdA promoter (*PgpdA*), and the *trpC* terminator (Ttrpc) for fungal transformation (Wang et al., 2021). ORF1-4 were amplified and cloned into the backbone vector, resulting in pDHt-SK-bar-PgpdA-ORF1-4. Four constructs were transformed into fungal cells, and insertion was confirmed using RT-PCR and qRT-PCR. The transformed mutants were named Vf/OE1-4.

### Phenotypic assays

For phenotypic assays, three replicates per strain were conducted to examine possible differences between MaPmV1-infected and MaPmV1-free strains (Vi and Vf), as previously described (Wang et al., 2019).

For the vegetative growth assays, 1 μL of a conidial suspension with a concentration of 1 × 10^7^ conidia/mL of Vi and Vf strains was centrally spotted on PDA, SDAY, and 1/4SDAY plates (amended with 1/4 of the nutrients in SDAY) and cultivated for 14 days. The fungal colonies were then photographed, and the diameter of each colony was measured (Wang et al., 2019).

To assess conidiation capacity, 30 μL of a conidial suspension with a concentration of 1 × 10^7^ conidia/mL of Vi and Vf strains were evenly spread on PDA plates (35 mm in diameter) and cultured for 14 days in the dark. These samples were then placed in 30 mL of 0.05% Tween 80 and conidia were released by vortex-mixing. The conidial concentration was determined using a hemocytometer and converted to a conidial yield per unit area of plate culture (*N* conidia/cm^2^) (Wang et al., 2018).

To assess the tolerance of the Vi and Vf strains to various chemicals, 1 μL of conidial suspension with a concentration of 1 × 10^7^ conidia/mL was centrally spotted on PDA plates containing NaCl (1 M), H_2_O_2_ (7 mM), or Congo red (2 mg/mL) and cultured for 14 days. The diameter of each colony was measured and photographed to determine the relative inhibition rate.

To evaluate the germination rate of the Vi and Vf strains, 10 μL of conidial suspension with a concentration of 5 × 10^6^ conidia/mL was dropped onto the center of PDA plates. The germination rate of conidia on each plate was evaluated by microscope (Olympus BX 51, Tokyo, Japan), counting every 2 h from 4 h onwards (Wang et al., 2014). At least 300 conidia per plate were counted to calculate the germination rates, and the SPSS software was used to calculate the median germinate time (GT_50_).

To examine the conidial thermotolerance and ultraviolet B (UV-B) irradiation resistance of each strain, conidial samples were exposed to a hot water bath at 45°C for 1.5 h and to UV-B irradiation (312-nm wavelength at 100 μJ/cm^2^) by HL-2000 Hybrilinker (UVP, CA, United States), respectively (Yao et al., 2010). After 24 h of culture, the germination of conidia was observed under a microscope and the relative germination rates were calculated (Wang et al., 2019).

For virulence assays, bioassays with fifth-instar *Galleria mellonella* larvae were carried out as previously described (Tong et al., 2020; Xie et al., 2020). Briefly, larvae were either dipped into conidial suspension (1 × 10^7^ conidia/mL) for 90 s or injected with 10 μL of conidial suspension (1 × 10^5^ conidia/mL) into the hemocoel. Each group contained 20 larvae, and each treatment was repeated three times. The mortality of larvae was recorded every 12 h, and the median lethal time (LT_50_) was calculated using the SPSS software.

### Quantitative RT-PCR (qRT-PCR)

To analyze the transcript levels of genes related to growth, conidiation, and DNA damage repair, the Vi and Vf1 strains were cultured on PDA plates with sterile cellophane for 14 days, SDAY plates with sterile cellophane for 2.5 days, and cultured on PDA plates with sterile cellophane for 24 h after treatment of UV-B irradiation, respectively. Total RNA was extracted using TRIzol reagent (Invitrogen, Foster City, CA, United States), and cDNAs were synthesized using the HiScript III 1st strand cDNA synthesis kit (Vazyme, Nanjing, China). Quantitative RT-PCR (qRT-PCR) was performed on a CFBR96TM Real-Time PCR System (Bio-Rad, Hercules, CA, United States) using the AceQ qPCR SYBR Green Master Mix (Vazyme, Nanjing, China). The glyceraldehyde-3-phosphate dehydrogenase (*gpd*) gene was used as an internal control, and the relative transcript levels were calculated using the 2^-ΔΔCT^ method (Livak and Schmittgen, 2001; Fang and Bidochka, 2006).

### Statistical analysis

All data were obtained from at least three independent biological replicates, and statistical analyses were performed using GraphPad Prism version 7.0 and SPSS v23.0 software (SPSS Inc., Chicago, IL, United States). In the statistical analysis of the biological character experiments, we employed Student’s t-test to compare the data (mean ± SE) between the Vi and Vf isolates. Additionally, for the comparison between Vi, Vf isolates and Vf/OE1-4, we utilized one-way analysis of variance (ANOVA) followed by appropriate multiple comparison procedures. A *value of p* of less than 0.05 was considered statistically significant.

## Results

### Sequencing and molecular characterization of MaPmV1 in *M. anisopliae* isolate RCEF3284

**Figure 1:**
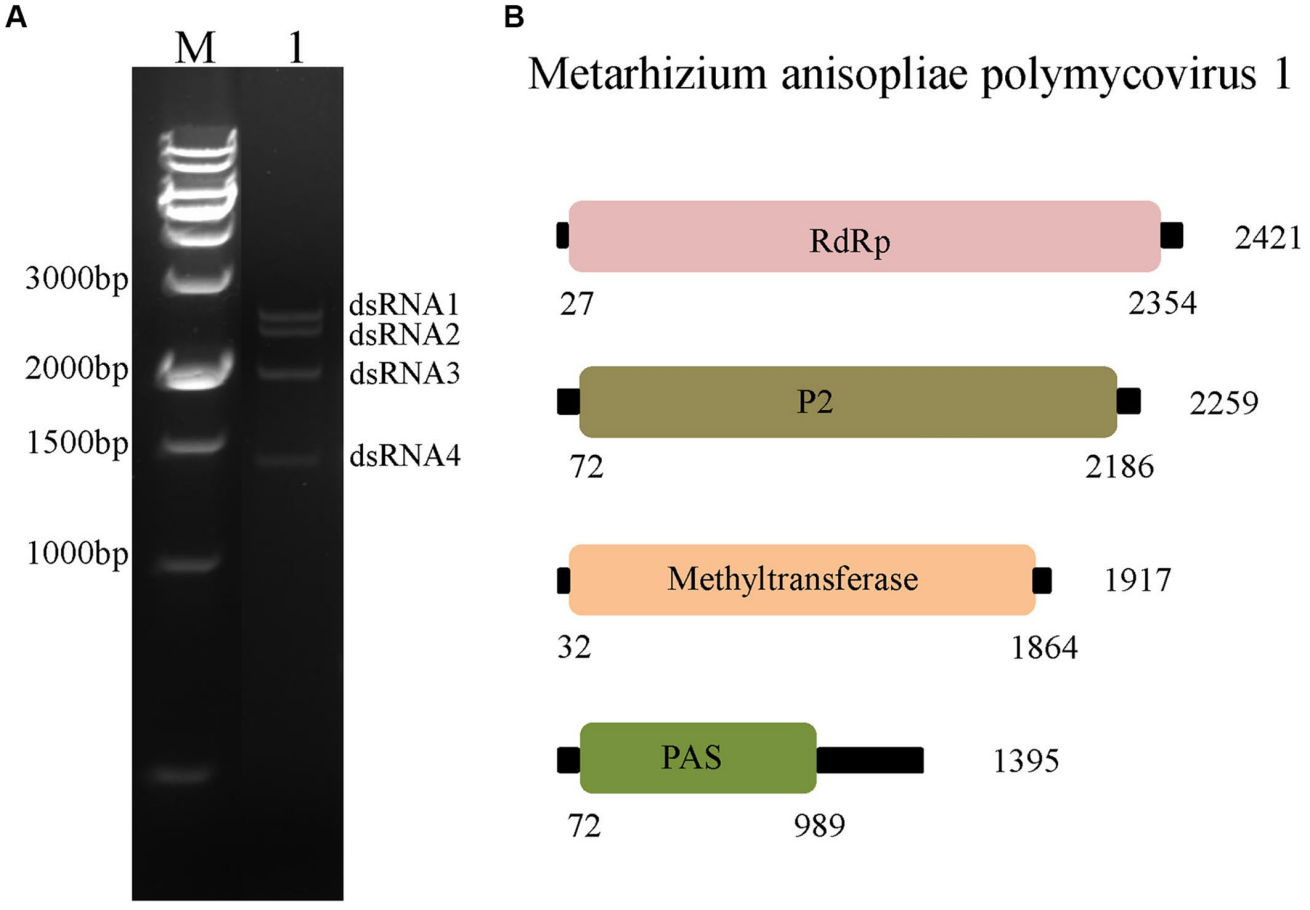
Characterization of Metarhizium anisopliae polymycovirus 1 (MaPmV1). **(A)** Characterization of MaPmV1. Lane M: DNA molecular weight marker; Lane 1: dsRNAs of MaPmV1. **(B)** Schematic representation of the genomic organization of MaPmV1.

After phenol/chloroform extraction and cellulose isolation, four dsRNA segments were detected in MaPmV1 from *M. anisopliae* isolate RCEF3284. These segments were named dsRNA 1 (∼2.4 kbp), dsRNA 2 (∼2.3 kbp), dsRNA 3 (∼1.9 kbp), and dsRNA 4 (∼1.4 kbp) in decreasing order of nucleotide length (Figure 1A). The complete sequences of dsRNA1–4 were determined using metagenomic sequencing, RT-PCR, and RACE cloning, and have been deposited in the GenBank database under accession numbers OP627094–627097, respectively.

**Figure 2:**
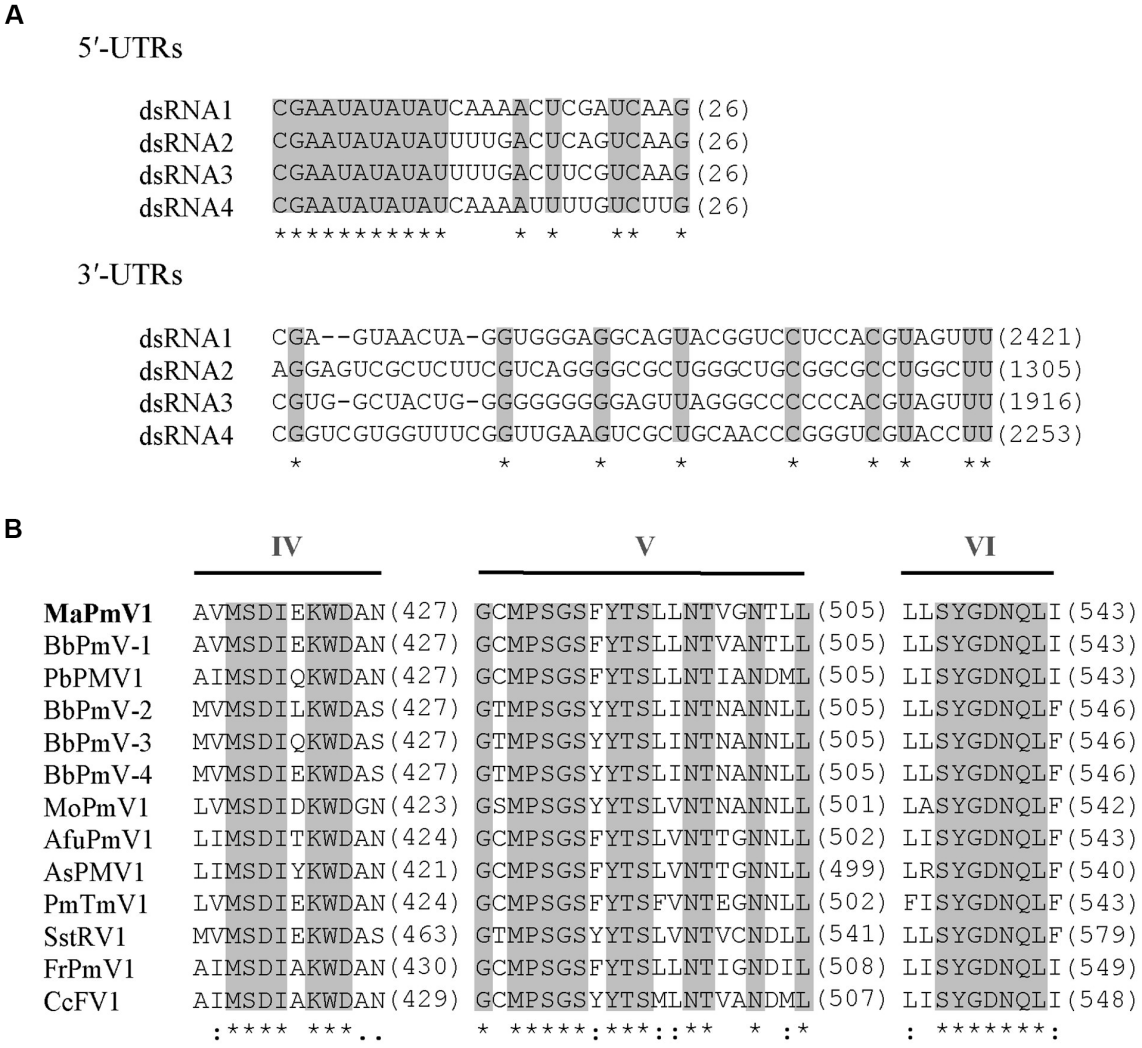
Alignment and sequence similarity analysis of MaPmV1. **(A)** Alignment of the 5′ and 3′ terminal nucleic acid sequences of MaPmV1. Shaded areas represent 100% nucleotide identity. **(B)** Multiple amino acid sequence alignment of the putative RdRp of MaPmV1 and other polymycoviruses (Supplementary Table S1). Shaded areas indicate a position with a single, fully conserved residue.

The full-length cDNA sequences of segments 1–4 were analyzed and found to be 2,421, 2,259, 1917, and 1,395 bp, respectively, with GC content of 58.5, 59.8, 58.1, and 63.8%, respectively. Each of the dsRNA segments was predicted to contain a single ORF and designated as ORF1, ORF2, ORF3, and ORF4, respectively (Figure 1B). However, the first 11 base pairs of the 5’-UTRs in the four dsRNAs are highly conserved (CGAAUAUAUAU), while the 3’-UTRs have a potential conserved element but differ in length (Figure 2A).

Blastx search revealed that the four genomic segments encode proteins with 56.3–70.0% amino acid identity to the counterparts of Beauveria bassiana polymycovirus 1 (BbPmV-1), a member of the genus *Polymycovirus*. Based on the genomic organization, UTR comparison, and Blastx search, the mycovirus with four dsRNA elements from *M. anisopliae* strain RCEF3284 is proposed as a novel polymycovirus and tentatively named Metarhizium anisopliae polymycovirus 1 (MaPmV1).

MaPmV1-ORF1 encodes an RNA-dependent RNA polymerase (RdRp) of 775 amino acids (aa) with a molecular mass of approximately 86.10 kDa. It shares the closest resemblance with the RdRp (YP_009352879.1) of BbPmV-1 with a sequence identity of 69.55, 100% coverage, and an E-value of 0 (Kotta-Loizou and Coutts, 2017). The MaPmV1 RdRp belongs to the protein family RdRP_1 (Pfam 00680), and contains three conserved motifs (IV-VI) with a GDNQ motif typically found in the RdRp of -ssRNA viruses of *Mononegavirales* (Figure 2B).

MaPmV1-ORF2 (nt 72–2,186) encodes a protein of unknown function (P2) with a length of 704 aa and a molecular mass of 75.40 kDa. BLASTp search revealed a sequence identity of 70.03% with BbPmV-1 (YP_009352876.1), 100% coverage, and an E-value of 0.

MaPmV1-ORF3 is predicted to encode a protein of 610 aa (Mr, 66.62 kDa) and spans from nucleotide positions 32 to 1864. The protein shows similarity to methyltransferase of BbPmV-1 (YP_009352877.1) with a sequence identity of 56.32, 99% coverage, and an E-value of 0. It also contains a methyltransferase domain (Methyltransf_25; Pfam13649) from amino acid residue position 123 to position 201.

The fourth ORF of MaPmV1 encodes a potential proline-alanine-serine rich protein (PASrp) of 305 aa with a molecular mass of 32.16 kDa. BLASTp search showed a sequence identity of 69.38% with a BbPmV-1 sequence (YP_009352878.1), 99% coverage, and an E-value of 5e-138.

### Phylogenetic analysis of MaPmV1

**Figure 3:**
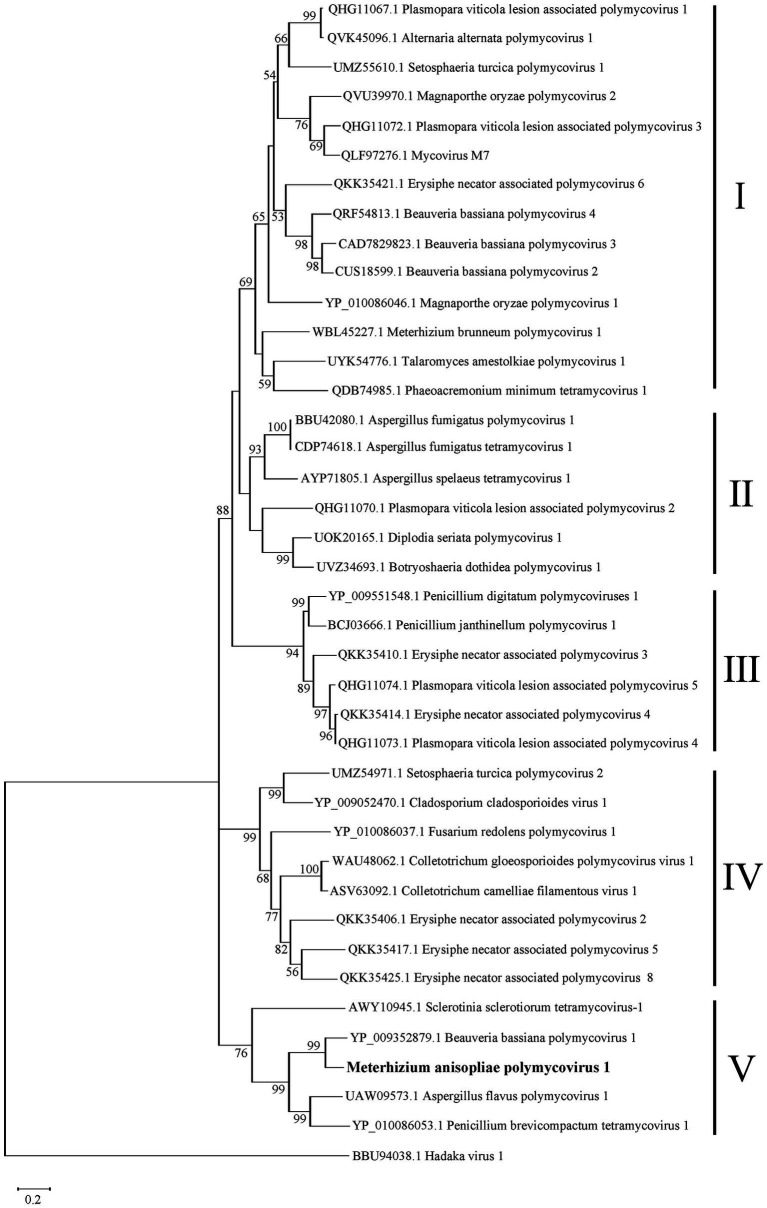
Phylogenetic analysis of MaPmV1. The maximum likelihood (ML) phylogenetic tree is based on RdRp sequences of MaPmV1 along with 38 other polymycoviruses, with Hadaka virus 1 serving as the outgroup.

To clarify the phylogenetic relationship of MaPmV1 with other members in the *Polymycoviridae*, we constructed a phylogenetic tree based on the amino acid sequences of the RNA-dependent RNA polymerase (RdRp) from thirty-eight known polymycoviruses, including five mycoviruses from entomopathogenic fungi, with Hadaka virus 1 as the outgroup (Figure 3). The phylogenetic tree revealed that these polymycoviruses were divided into five clades, four of which had well-supported values. Furthermore, MaPmV1 fell into clade V, of which all members have four dsRNA segments, and was found to be most closely related to BbPmV-1 from *B. bassiana* with a bootstrap support value of 99%. Additionally, the other four polymycoviruses from entomopathogenic fungi were grouped into clade I, and Metarhizium brunneum polymycovirus 1 was closely grouped with Phaeoacremonium minimum tetramycovirus 1 and Talaromyces amestolkiae polymycoviruses.

### Generation of MaPmV1-infected and-free isogenic lines

*M. anisopliae* isolate RCEF3284 was cured of MaPmV1 through single conidium isolation. A total of 24 single-spore isolates derived from the parent strain were screened for the presence of dsRNA segments (Supplementary Figure S1A), and only one isolate was found to contain MaPmV1. This suggests that MaPmV1 is not efficiently transmitted to the asexual progeny. The elimination of MaPmV1 was further confirmed using sequence-specific oligonucleotide primers RdRpF and RdRpR (Supplementary Table S3) in RT-PCR (Supplementary Figures S1B,C).

### MaPmV1 increases vegetative growth and conidiation of *M. anisopliae*

**Figure 4:**
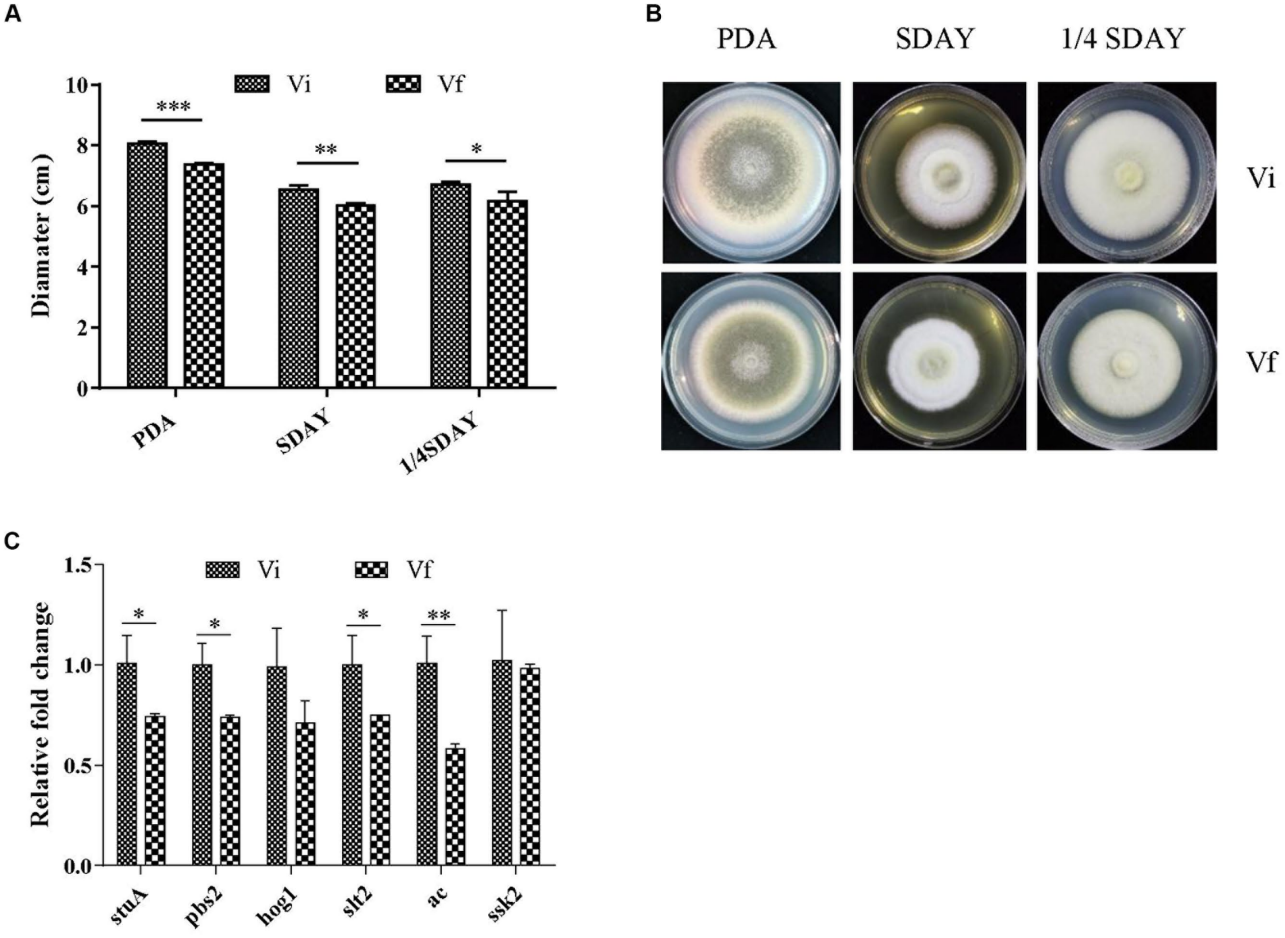
Effect of MaPmV1 on growth of *M. anisopliae*. **(A)** Growth diameter of different strains cultured on PDA, SDAY, and 1/4SDAY medium for 14 d. Student’s *t*-test to compare the data. *, *p* < 0.05; **, *p* < 0.01; ***, *p* < 0.001. **(B)** Colony morphology of different strains cultured on PDA, SDAY, and 1/4SDAY plates (90 mm) for 14 d. **(C)** Relative expression levels of growth-related genes as shown by qRT-PCR. Statistical significance was determined by Student’s *t*-test. *, *p* < 0.05; **, *p* < 0.01.

We investigated the effect of MaPmV1 infection on fungal development. MaPmV1-infected *M. anisopliae* strain RCEF3284 (Vi) and MaPmV1-free strain (Vf) were cultured on PDA, SDAY, and 1/4 SDAY for 14 days at 25°C. Vi colony diameters showed a significant increase of 8.68% (*p* < 0.001), 7.65% (*p* < 0.01), and 7.96% (*p* < 0.05) on SDAY, PDA, and 1/4 SDAY plates, respectively, compared to the Vf strain (Figures 4A,B). Moreover, transcript levels of four growth-related genes, *stuA*, *pbs2*, *slt2*, and *ac*, were significantly upregulated in Vi strains compared to Vf strains (Figure 4C). These results indicate that MaPmV1 enhanced the growth rate by upregulated the expressions of growth-related genes of the host.

**Figure 5:**
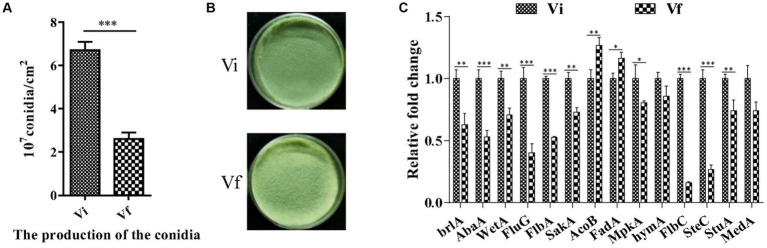
Effect of MaPmV1 on conidiation of *M. anisopliae*. **(A)** Conidiation of different strains cultured on PDA plates for 14 d. **(B)** Student’s *t*-test to compare the data. ***, *p* < 0.001. Colony morphology of different strains cultured on PDA plates (35 mm) for 14 d. **(C)** Relative expression levels of conidiation-related genes as shown by qRT-PCR. Statistical significance was determined by Student’s *t*-test. *, *p* < 0.05; **, *p* < 0.01; ***, *p* < 0.001.

We assessed *M. anisopliae* conidiation on solid media 14 days post-inoculation (dpi). The conidial yields of Vi and Vf were 6.71 ± 0.22 and 2.61 ± 0.17 (10^7^ conidia/cm^2^), respectively (Figures 5A,B). The conidial yield of the Vi strain was significantly (2.6-fold) higher than that of the Vf strain. Furthermore, we measured the expression of 14 genes involved in conidiation of filamentous fungi, and found that 11 conidiation-related genes, including three key regulators in asexual development (*brlA*, *abaA*, and *wetA*), were significantly upregulated in the MaPmV1-infected strain compared to the MaPmV1-free strain (Figure 5C).

### MaPmV1 does not affect host thermal tolerance, but increases fungal sensitivity to UV-B irradiation

We first evaluated the effect of MaPmV1 on the conidial germination of the host fungus. We found no significant differences in the median conidial germination time (GT_50_) between Vi and Vf strains (Supplementary Figure S2).

**Figure 6:**
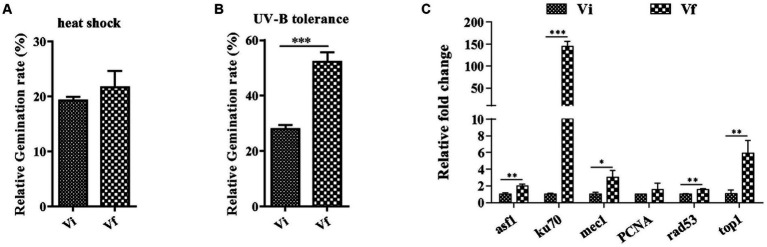
Effect of MaPmV1 on heat shock and UV-B irradiation tolerance. **(A)** Comparison of relative germination rates of *M. anisopliae* strains following heat shock treatment for 24 h. **(B)** Comparison of relative germination rates of *M. anisopliae* strains following UV-B irradiation treatment for 24 h. Student’s *t*-test to compare the data. ***, *p* < 0.001. **(C)** Comparison of relative expression levels of DNA damage repair genes between different strains as shown by qRT-PCR. Statistical significance was determined by Student’s *t*-test. *, *p* < 0.05; **, *p* < 0.01; ***, *p* < 0.001.

Next, we investigated whether MaPmV1 affects the host fungus’s tolerance to environmental stresses. We exposed conidia to heat or UV-B irradiation stress and calculated the conidial germination rates after 24 h. We found no significant differences in conidial germination rates between Vi and Vf strains following heat shock (Figure 6A). However, after UV-B irradiation treatment, the conidial germination rates of the MaPmV1-infected strain were significantly decreased compared to the MaPmV1-free strains, showing a 46.65% decrease in UV-B tolerance (Figure 6B). Moreover, we found that transcript levels of five out of six DNA damage repair genes (histone deposition protein Asf1 (*asf1*), Ku70 protein (*ku70*), phosphatidyl inositol 3-kinase (*mec1*), serine/threonine-protein kinase domain protein (*Rad53*), and topoisomerase I (*top1*)) were significantly down-regulated in the Vi strain (Figure 6C).

We also examined whether MaPmV1 affects the host’s sensitivity to chemical stresses, including osmotic stress, cell wall integrity, and oxidative stress. We found that MaPmV1 did not affect the host’s sensitivity to these chemical stresses (Supplementary Figure S4).

### MaPmV1 has no impact on the virulence of *M. anisopliae*

**Figure 7:**
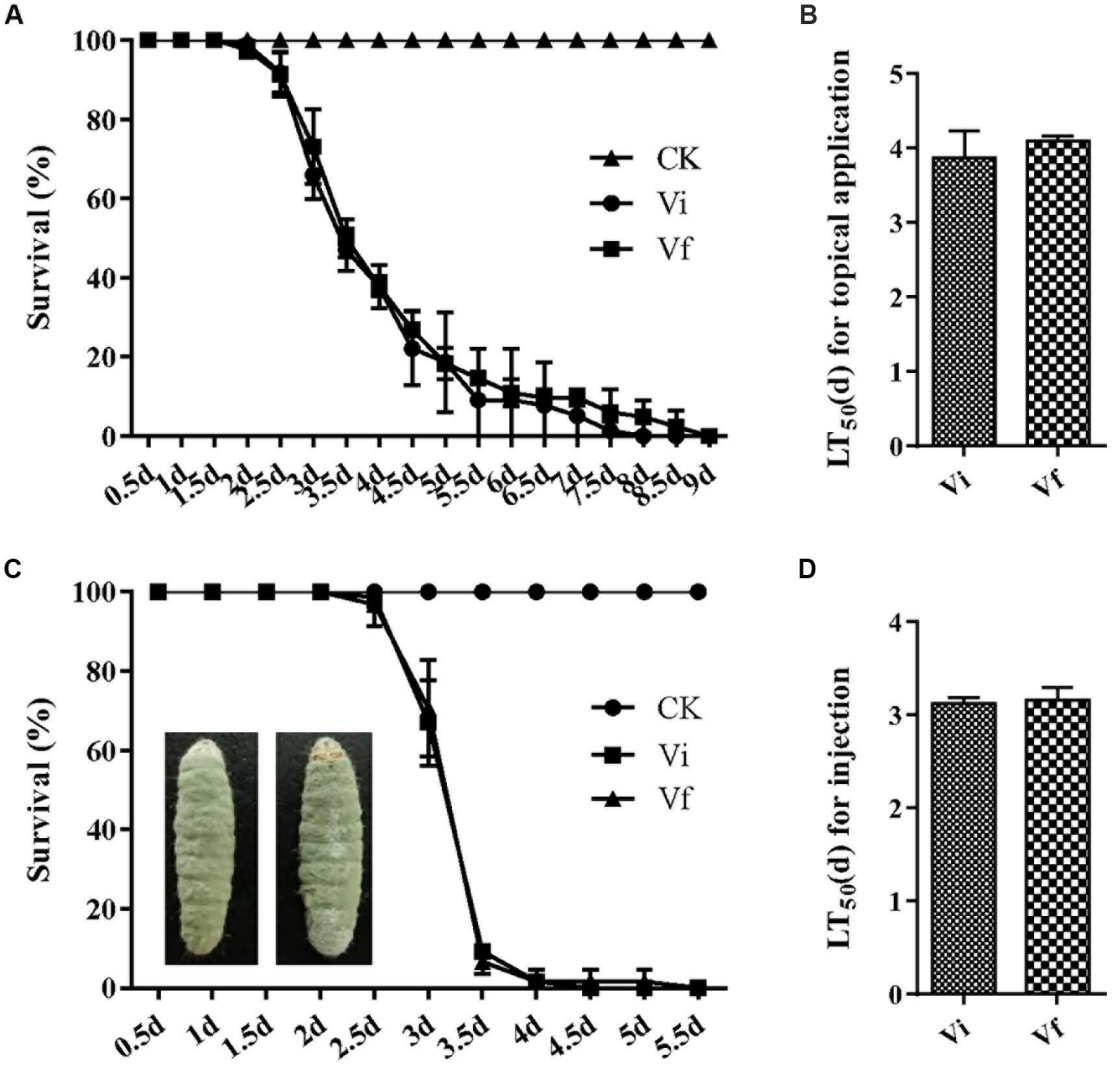
Effect of MaPmV1 on fungal virulence. **(A)** Survival rates of *G. mellonella* larvae after topical infection with conidial suspensions from different strains. Control insects were treated with sterile water. **(B)** Mean lethal times (LT_50_) of different strains after topical application. Statistical significance was determined by Student’s *t*-test. **(C)** Survival rates of *G. mellonella* larvae after intrahemocoel injection with conidial suspensions from different strains. Control insects were treated with sterile water. **(D)** Mean lethal times (LT_50_) of different strains after injection. Statistical significance was determined by Student’s *t*-test.

To assess the impact of MaPmV1 on the virulence of *M. anisopliae*, we employed *G. mellonella* as a model infection system. However, we did not observe any significant difference in virulence between the Vi and Vf strains in both topical application (with LT_50_ values of 3.87 ± 0.21 and 4.10 ± 0.04 d, respectively) and intrahemocoel injection (with LT_50_ values of 3.13 ± 0.03 and 3.15 ± 0.08 d, respectively), and external conidia are massively produced on the mycosed insect cadaver (Figure 7). These results indicate that MaPmV1 has no impact on the virulence of the host.

### Each ORF of MaPmV1 does not cause any phenotypic changes in *M. anisopliae*

**Figure 8:**
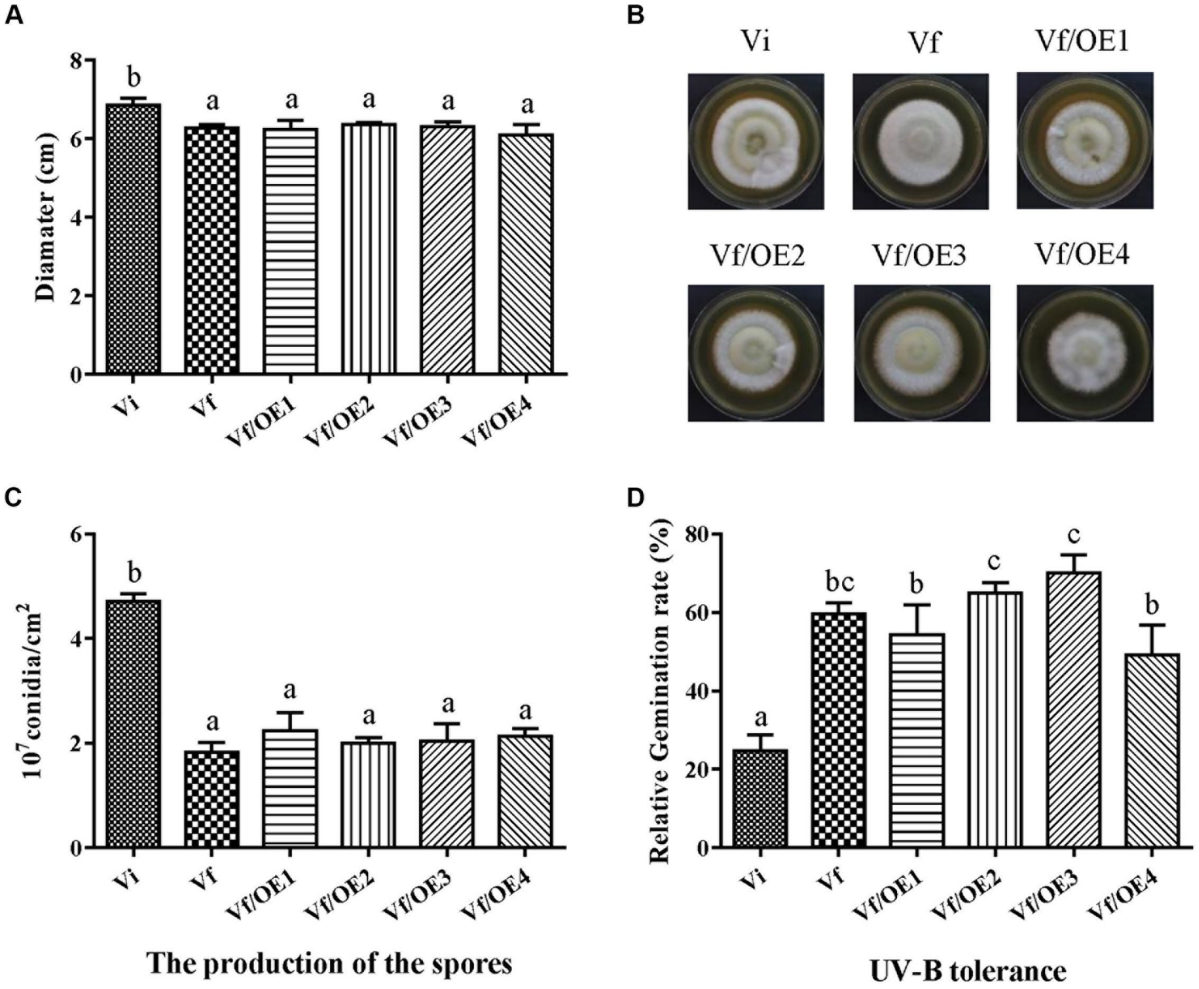
Effect of MaPmV1 ORF1-4 expression on growth, conidiation, and UV-B irradiation tolerance of *M. anisopliae*. **(A)** Growth diameter of different strains cultured on SDAY medium for 14 d. Statistical significance was determined by one-way analysis of variance. Different letter indicates a significant difference at *p* < 0.05. **(B)** Colony morphology of different strains cultured on SDAY plates (90 mm) for 14 d. **(C)** Conidiation of different strains cultured on PDA plates for 14 d. Statistical significance was determined by one-way analysis of variance. Different letter indicates a significant difference at *p* < 0.05. **(D)** Germination rates of different strains following UV-B irradiation treatment for 24 h. Statistical significance was determined by one-way analysis of variance. Different letter indicates a significant difference at *p* < 0.05.

To identify which individual gene(s) of MaPmV1 influence the host phenotype, we created over-expressing strains (Vf/OE1-4) of MaPmV1 ORF1-4 using ATMT and confirmed their expression through RT-PCR and qRT-PCR (Supplementary Figure S5). No significant changes in conidial yields were observed among strains Vf, Vf/OE1, Vf/OE2, Vf/OE3, and Vf/OE4, with counts of 1.83 ± 0.11, 2.23 ± 0.20, 2.00 ± 0.06, 2.04 ± 0.19, and 2.13 ± 0.08 (1 × 10^7^ conidia/cm^2^), respectively (Figure 8C). Similar results were observed regarding sensitivity to UV-B irradiation (Figure 8D). Thus, we concluded that individual MaPmV1 ORF1-4 do not induce any phenotypic changes in *M. anisopliae* (Figures 8A,B).

## Discussion

*Polymycovirus* is a type of mycovirus that lies between dsRNA and + ssRNA viruses in addition to encapsidated and capsidless RNA viruses. The members of the *Polymycoviridae* family possess a multipartite genome that ranges from 7.2–12.5 kbp and infect both ascomycetes and basidiomycetes (Jia et al., 2017; Kotta-Loizou and Coutts, 2017). In this study, we have identified a novel polymycovirus, MaPmV1, which is the first polymycovirus isolated from ascomycetes *M. anisopliae*. Like most polymycoviruses, MaPmV1 has a genome of 8.0 kbp consisting of four dsRNA segments, each containing a single ORF (Figure 1). MaPmV1-dsRNA1 encodes an RdRp with three conserved motifs (VI-IV) that are related to virus replication (Filippou et al., 2021). The function of the putative protein encoded by the second dsRNA of MaPmV1 remains unknown, as is the case with most known polymycoviruses (Mahillon et al., 2019; Gao et al., 2021). The ORF of MaPmV1-dsRNA 3 encodes a protein with methyltransferase activity, which is conserved in the alphavirus-like superfamily and is responsible for adding a guanylyl residue to the 5′ end of dsRNAs to form a G (5’) ppp (5’)N cap structure (Fujimura and Esteban, 2011; Kanhayuwa et al., 2015). The last dsRNA of MaPmV1 encodes a PAS-rich protein containing an over-representative number of proline (P), alanine (A), and serine (S) residues, which is proposed to protect the genomic RNA similar to viral capsids (Sato et al., 2020).

In this study, we have provided the first evidence of the effect of a polymycovirus on *Metarhizium* fungi. Specifically, we have shown that MaPmV1 significantly increases the growth rate of *M. anisopliae*, and the expression of growth-related genes significantly upregulated in MaPmV1 infected isolate. The genes were confirmed related to the enhancement of growth in other *Metarhizium* isolates rather than *M. anisopliae* (Wang et al., 2014; Song et al., 2016; Wang et al., 2018; Yang et al., 2018; Tong et al., 2020), which may limit the universality of our results. Similar to the effect observed for other entomopathogenic fungus *B. bassiana* and human pathogenic fungus *Aspergillus fumigatus* when infected with polymycoviruses such as BbPmV-3 and *A. fumigatus* A78 isolate, respectively (Ozkan and Coutts, 2015; Kotta-Loizou and Coutts, 2017; Filippou et al., 2021). However, it should be noted that not all mycoviruses have a positive effect on their hosts, as Colletotrichum camelliae filamentous virus 1 (CcFV-1) has been shown to impair host growth and cellular homeostasis (Jia et al., 2017).

Multiple studies have shown that entomopathogenic fungus strains infected with polymycoviruses can increase their conidiation capacity. For instance, studies on BbPmV-1 and BbPmV-3 infected strains indicate a two-fold increase in sporulation compared to virus-free isogenic lines (Filippou et al., 2021). Similarly, virus-infected *Beauveria bassiana* strains show a 96% increase in conidial yield compared to virus-free strains, which repress the expression of the key conidiation-related genes *abaA* and *brlA* (unpublished). Our study also observed a similar phenomenon, whereby MaPmV1 significantly enhances the conidiation capacity of the host and upregulates the expression of conidiation-associated genes, including three key regulators (*brlA*, *abaA*, and *wetA*) (Figure 5).

However, while MaPmV1 enhances the fitness of *M. anisopliae* in fungal development, it also decreases its fitness by increasing sensitivity to UV-B irradiation. Vi strain downregulates the transcript levels of DNA damage repair genes, including *asf1*, *mec1*, *rad53*, and *top1*, while increasing their expression levels by approximately 2-, 3-, 1.5-, and 6-fold, respectively. The finding that *Metarhizium* virus increases the sensitivity of *M. majus* to UV-B irradiation aligns with the observed effect of partitivirus on *M. majus* (unpublished).

Furthermore, it is worth noting that heterologous expression of viral proteins may lead to the alteration of morphology and growth (Urayama et al., 2014). Thus, we obtained MaPmV1 ORF1-4 expressing strains (Vf/OE1-4) in Vf isolate. However, the four single viral protein-expressing strains (Vf/OE1-4) exhibited similar colony morphologies and conidiation as the wild-type. While the sensitivity to UV-B irradiation varied to some extent among Vf/OE1-4, there was no significant difference between Vf/OE1-4 and Vf in this regard. Similar results were observed with Fusarium graminearum virus 1 (FgV1) (Yu et al., 2020). These results suggest that the modulation of mycoviruses to the biological characteristics of their host is not always dependent on the effect of a single virus gene product.

We obtained multiple virus-free strains from the virus-positive strains and performed our experiments using one of these strains. We acknowledge the potential risk of mutations in the host genome during the acquisition of virus-free strains. Thus, using only one strain for phenotype analysis may limit the generalizability of our findings and could introduce potential biases.

In summary, we have characterized a novel mycovirus, MaPmV1, isolated from the entomopathogenic fungus *M. anisopliae*, which is related to the members of the *Polymycoviridae* family. This is the first report of a polymycovirus in *M. anisopliae*. Furthermore, the infection of MaPmV1 leads to increased growth rate, sporulation, and sensitivity to UV-B irradiation of the host. This study suggests that mycoviruses can enhance the fungal development in entomopathogenic fungi and could be exploited for the large-scale improvement of conidia production.

## Data availability statement

The datasets presented in this study can be found in online repositories. The names of the repository/repositories and accession number(s) can be found in the article/Supplementary material.

## Author contributions

BH conceived and designed the study, edited the manuscript, and supervised the project. PW and GY wrote the manuscript, conducted the experiments, and analyzed the data. HL done a part of the experiments. All authors contributed to the article and approved the submitted version.

## Funding

This work was supported by the National Natural Science Foundation of China (grant No. 32172473).

## Conflict of interest

The authors declare that the research was conducted in the absence of any commercial or financial relationships that could be construed as a potential conflict of interest.

## Publisher’s note

All claims expressed in this article are solely those of the authors and do not necessarily represent those of their affiliated organizations, or those of the publisher, the editors and the reviewers. Any product that may be evaluated in this article, or claim that may be made by its manufacturer, is not guaranteed or endorsed by the publisher.

## Footnotes

^1^ https://www.ncbi.nlm.nih.gov/orffinder
