# Supplementary material for: Infection with a novel polymycovirus enhances growth, conidiation and sensitivity to UV-B irradiation of the entomopathogenic fungus Metarhizium anisopliae
Source: Front Microbiol. 2023 Jul 4;14:1214133. doi: 10.3389/fmicb.2023.1214133 (PMC10352681; doi:10.3389/fmicb.2023.1214133)
Supplement: Supplementary file 1 [file Data_Sheet_1.docx]

***Supplementary Materials***

**Infection with a Novel Polymycovirus Enhances Growth, Conidiation and Sensitivity to UV-B Irradiation of the Entomopathogenic Fungus *Metarhizium anisopliae***

Ping Wang^1^ Guogen Yang^1, 2^ Hanwen Lu^1^ Bo Huang^1,*^

^1^ Anhui Provincial Key Laboratory of Microbial Pest Control, Anhui Agricultural University, Hefei 230036, China

^2^School of Plant Protection, Anhui Agricultural University, Hefei 230036, China

^*^Corresponding authors.

Anhui Provincial Key Laboratory of Microbial Pest Control, Anhui Agricultural University, Hefei 230036, China.

Tel./ Fax: +86-551- 65786211.

E-mail address: bhuang@ahau.edu.cn (Bo Huang)


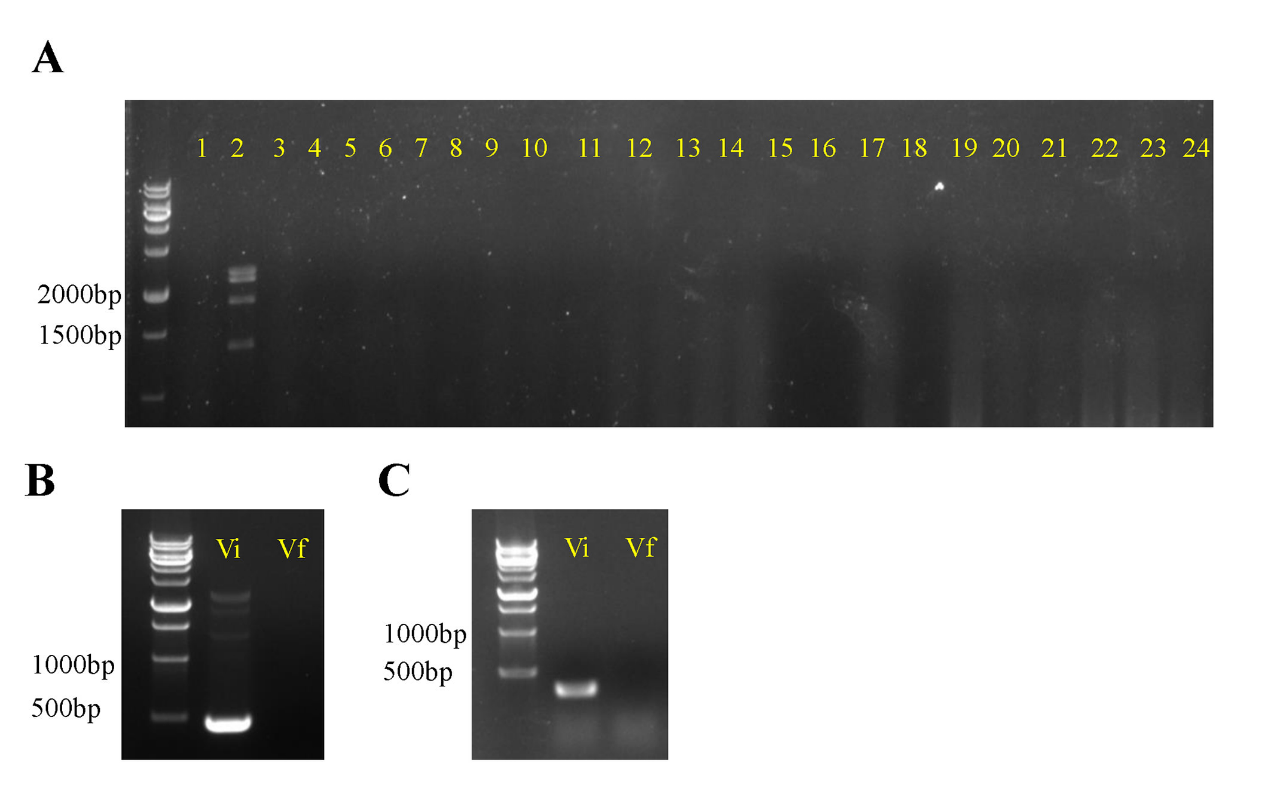


**Supplementary Figure 1 Vertical transmission of MaPmV1.**

**(A)** dsRNAs extraction from single spore of RCEF3284.

**(B)** RT-PCR of dsRNA with MaPmV1 specific primers RdRpF and RdRpR (Supplementary Table 3).

**(C)** RT-PCR of total RNA with MaPmV1 specific primers RdRpF and RdRpR (Supplementary Table 3).


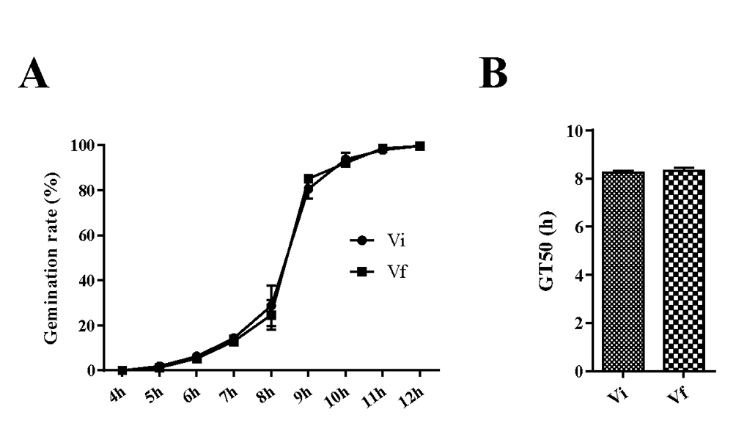


**Supplementary Figure 2 Effect of different strains on germination of *M. anisopliae*.**

**(A)** The germination rate of different strains.

**(B)** The median germination time (GT_50_) of different strains.


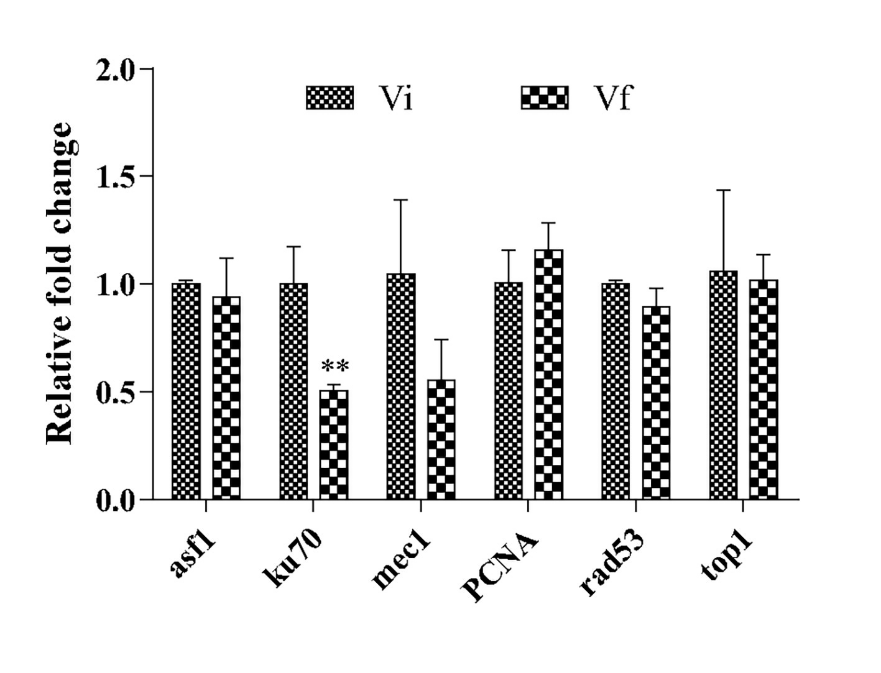


**Supplementary Figure 3** Comparison of relative expression levels of DNA damage repair genes between different strains as shown before UV-B irradiation treatment by qRT-PCR. ANOVA **, P <0.01.


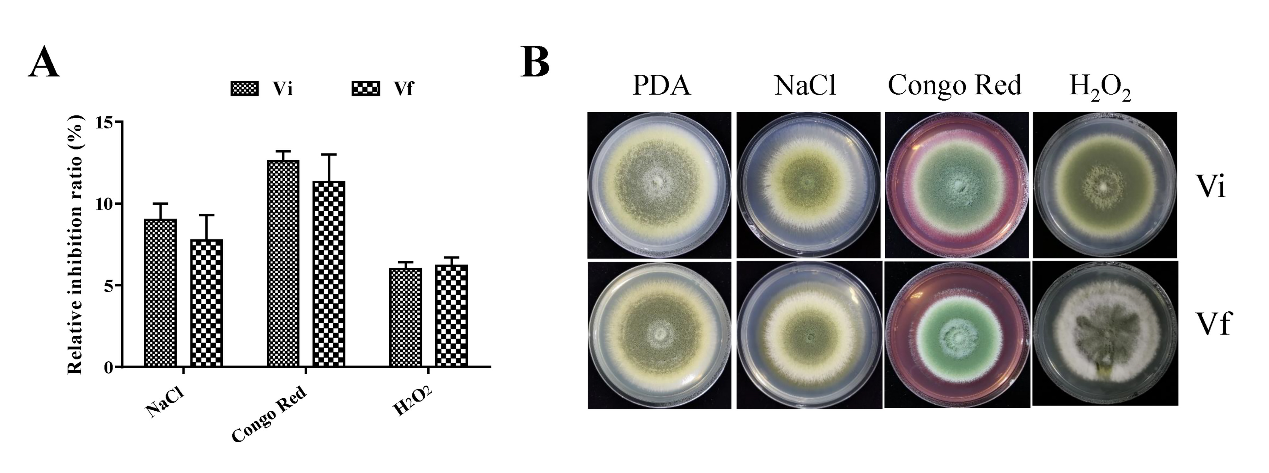


**Supplementary Figure 4 Effect of MaPmV1 on chemical stresses of *M. anisopliae*.**

**(A)** Growth diameter of different strains cultured on NaCl, Congo Red and H_2_O_2_ medium for 14 days.

**(B)** Colony morphology of different strains cultured on NaCl, Congo Red and H_2_O_2_ medium for 14 days.


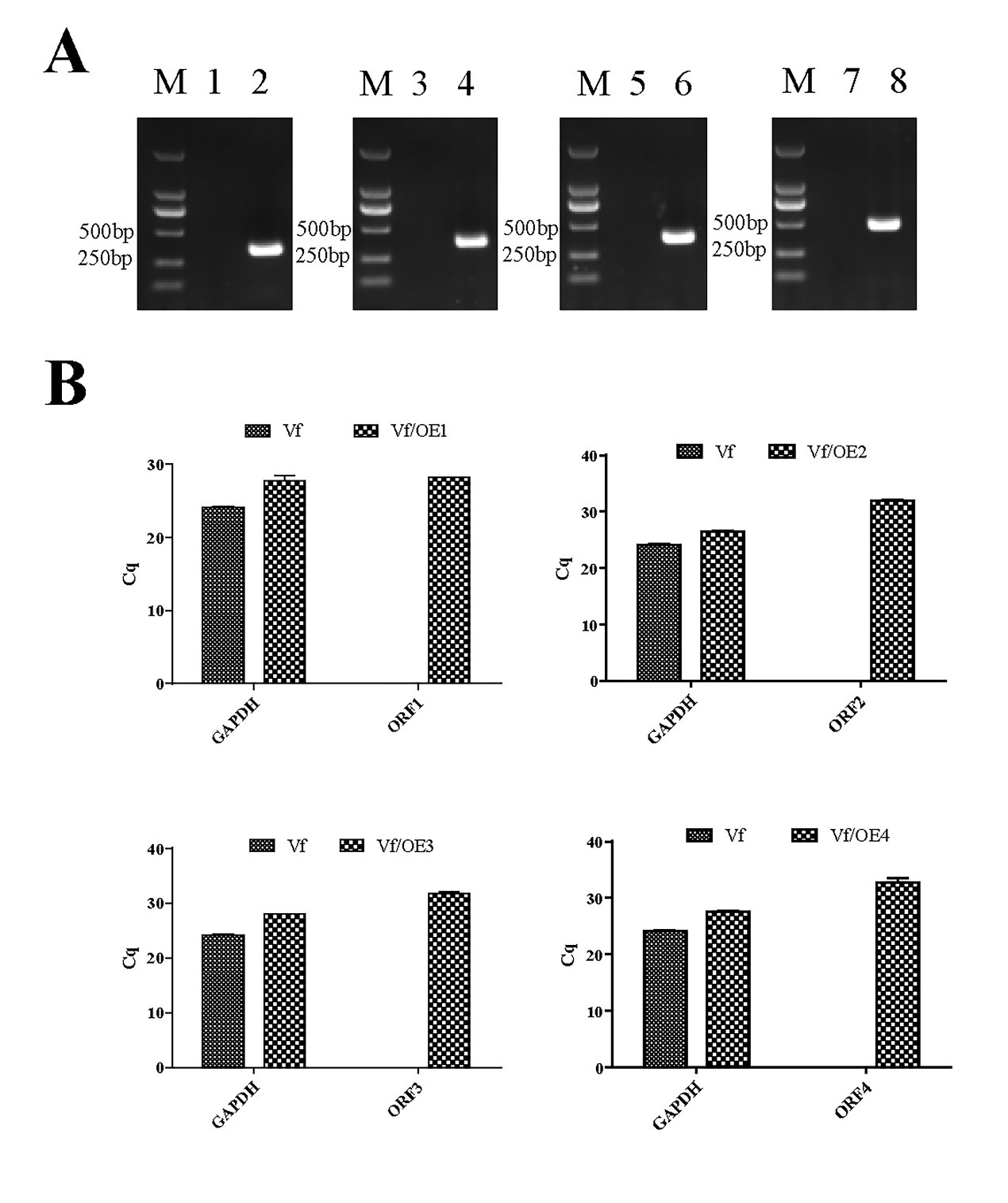


**Supplementary Figure 5 RT-PCR and qRT-PCR of Vf/OE1-4.**

**(A)** RT-PCR of Vf and Vf/OE1-4. M, DNA molecular weight marker; lane 1, RT-PCR of Vf using MaPmV1-ORF1 specific primers; lane 2, RT-PCR of Vf/OE1 using MaPmV1-ORF1 specific primers ORF1F, ORF1R; lane 3, RT-PCR of Vf using MaPmV1-ORF2 specific primers; lane 4, RT-PCR of Vf/OE2 using MaPmV1-ORF2 specific primers ORF2F, ORF2R; lane 5, RT-PCR of Vf using MaPmV1-ORF3 specific primers; lane 6, RT-PCR of Vf/OE3 using MaPmV1-ORF3 specific primers ORF3F, ORF3R; lane 7, RT-PCR of Vf using MaPmV1-ORF4 specific primers; lane 8, RT-PCR of Vf/OE4 using MaPmV1-ORF4 specific primers ORF4F, ORF4R. (The primers used in RT-PCR list in Supplementary Table 3,)

**(B)** The Cq values of Vf and Vf/OE1-4, GAPDH as the reference gene.

**Supplementary Table 1 Mycoviruses used in FIGURE 2B**

| **Virus name** | **Abbreviation** | **GenBank Acc.no.** |
| --- | --- | --- |
| Aspergillus fumigatus polymycovirus 1 | AfuPmV1 | BBU42080.1 |
| Aspergillus spelaeus tetramycovirus 1 | AsPMV1 | AYP71805.1 |
| Beauveria bassiana polymycovirus 1 | BbPmV-1 | YP_009352879.1 |
| Beauveria bassiana polymycovirus 2 | BbPmV-2 | CUS18599.1 |
| Beauveria bassiana polymycovirus 3 | BbPmV-3 | CAD7829823.1 |
| Beauveria bassiana polymycovirus 4 | BbPmV-4 | QRF54813.1 |
| Colletotrichum camelliae filamentous virus 1 | CcFV1 | ASV63092.1 |
| Fusarium redolens polymycovirus 1 | FrPmV1 | YP_010086037.1 |
| Magnaporthe oryzae polymycovirus 1 | MoPmV1 | YP_010086046.1 |
| Penicillium brevicompactum polymycovirus 1 | PbPMV1 | YP_010086053.1 |
| Phaeoacremonium minimum tetramycovirus 1 | PmTmV1 | QDB74985.1 |
| Sclerotinia sclerotiorum tetramycovirus-1 | SstRV1 | AWY10945.1 |

**Supplementary Table 2 Primers used for amplification of the terminal sequences of four dsRNA segments of Metarhizium anisopliae polymycovirus 1 (MaPmV1).**

|  | **Primer name** | **Sequences (5**'**-3**'**) of primers** |
| --- | --- | --- |
| dsRNA1 | dsRNA1L | GAAATTAAGGTCCTTCCGTCGC |
|  | dsRNA1R | GAATCCAGGACAAGGACCCACA |
| dsRNA2 | dsRNA2L | CAACCTGCTTGCTCGTGAGGTA |
|  | dsRNA2R | GTACGAAAGGCCATCTACAGCC |
| dsRNA3 | dsRNA3L | CTCTATCCCGTGAAGAAGACAC |
|  | dsRNA3R | TCGATAAGCGCGTGTCTAACAC |
| dsRNA4 | dsRNA4L | GCTTCCAATCCTCGTTGGACAG |
|  | dsRNA4R | CCGAGGATGCAAGACTTCGTTC |

**Supplementary Table 3 Primers used for RT-PCR of virus detection and expressing strains (Vf/OE1-4).**

|  | **Primer name** | **Sequences (5**'**-3**'**) of primers** |
| --- | --- | --- |
| **Primers used for RT-PCR of virus detection** | | |
| RdRp | RdRpF | CATCACCACAGTGTTCAAGGAG |
|  | RdRpR | TCATCGTCTTTCCTCCCATACA |
| ORF1 | ORF 1F | GCAAGAGGGAGCCGAAGA |
|  | ORF 1R | CGTCAAACGACAAGCGAATAA |
| ORF2 | ORF 2F | ATAACGTCACGGAATCCAGC |
|  | ORF 2R | CCTAAAGACCGCACCTCACTC |
| ORF3 | ORF 3F | CAGCCACCACGCCTATGA |
|  | ORF 3R | GCGGCACAACTCGAACAG |
| ORF4 | ORF 4F | ATCCTCGCTTACTCGTTCGTG |
|  | ORF 4R | AACCAGTCATCGTCGTTGTCC |

**Supplementary Table 4** **Paired primers used for transcriptional profiling of potential MaPmV1-targeted genes in *M. anisopliae* via qRT-PCR.**

| **Gene** | **Tag loci** | **Annotation** | **Sequences (5’-3’) of paired primers** |
| --- | --- | --- | --- |
| **Involved in growth** | | | |
| ssk2 | MANI_001632 | putative MAP kinase kinase kinase SskB | CGAAGACGCAGTGGAACA |
|  |  |  | ACCATTGGCATACGGAGC |
| pbs2 | MANI_013063 | mitogen activated protein kinase kinase | CCCGCCGACTCTTGCTACT |
|  |  |  | TCGCCATGACATCGTTGTTC |
| hog1 | MANI_028581 | stress-activated MAP kinase | TATTTCAAAGGCGGTTCA |
|  |  |  | GTATCTACAATGCCAGTCAG |
| stuA | MANI_024223 | APSES transcription factor | CCTTCGGTCCTACCACTT |
|  |  |  | CCCTCATCTTCCCACAAC |
| AC | H634G_06748 | Adenylate cyclase | GTCATCTTGAACTCCCTTGGC |
|  |  |  | GGCACCATACCGCAGACAC |
| slt2 | MAN_00887 | Serine/threonine-protein kinase domain protein | GCCTCAGCCATGCTAATC |
|  |  |  | CTCGCTCGTCTGGTTGTT |
| **Involved in conidial yield** | | | |
| brlA | MAN_03245 | transcription factor Fst12 | AAAGCCAGAAACCTTCAT |
|  |  |  | TACAAATCACCAGCGACA |
| AbaA | MAN_09977 | transcription factor AbaA | TGCCTTTACAGAGTTCCG |
|  |  |  | TTTGGGTATCTTTGTGAGC |
| WetA | H634G_00073 | hypothetical protein H634G_00073 | CCACGACCGACAATGACT |
|  |  |  | GAAACGCAGGATGAGACG |
| FluG | H634G_10199 | hypothetical protein H634G_10199 | TTTCGGAACAACTTGGATG |
|  |  |  | ATGTCGTCGTTCTTTAGCG |
| FlbA | MANI_110619 | regulator of G protein signaling pathway | CTCCGTCCGCAAGTTCCT |
|  |  |  | GGCTGAGAAGACTGGTGGC |
| SakA | MANI_028581 | stress-activated MAP kinase | TATTTCAAAGGCGGTTCA |
|  |  |  | GTATCTACAATGCCAGTCAG |
| AcoB | MANI_025870 | COP9 signalosome subunit 7 (CsnG) | CCCCTTATTGTATGGATCTACG |
|  |  |  | AGGAACGGCTGTGGTGGA |
| FadA | E5D57_002455 | Guanine nucleotide-binding protein subunit alpha | ACAGAGGAGAAGGAGGGC |
|  |  |  | CATACCGAGCAATAGCATC |
| MpkA | MANI_028514 | putative MAP kinase | GCCTCAGCCATGCTAATC |
|  |  |  | CTCGCTCGTCTGGTTGTT |
| hymA | MANI_026678 | conidiophore development protein hymA | GGAGTATGCCTAATGAGAAA |
|  |  |  | CAAGGAGGTACAGCAAATC |
| FlbC | MANI_001811 | C2H2 finger domain protein FlbC | GTTTCATCGTTTGGGATT |
|  |  |  | GAGAAGCAGAAGTGGCATA |
| SteC | MANI_005462 | MAP kinase kinase kinase Ste11 | TTCCGCAAGAATCACATCA |
|  |  |  | TTCCCATCTCCTTCAACACT |
| StuA | MANI_024223 | APSES transcription factor | TTCGGTCCTACCACTTCCT |
|  |  |  | TGCCCTCATCTTCCCACA |
| MedA | H634G_01238 | hypothetical protein H634G_01238 | AGCACCTAAGGATAGAAACG |
|  |  |  | GTGACTGGCAAGCAACAA |
| **Involved in DNA damage repair** | | | |
| asf1 | MANI_012796 | putative histone chaperone ASF1 | GCTACCTCGTGAGAACCA |
|  |  |  | GTCCAACCAGCAGTGAGT |
| ku70 | MANI_017242 | ku70 protein | GATTGGAAGAGGGAGGAC |
|  |  |  | GCATCTTTCTGTGCCTTG |
| mec1 | MAN_10382 | phosphatidyl inositol 3-kinase | GAGGCACTGAGCCACTTT |
|  |  |  | TCCAATTCCTGGAACAAA |
| PCNA | MAN_05117 | Proliferating cell nuclear antigen, PCNA | TCTGATTCACGCTCTGCC |
|  |  |  | CCTCGACGACCTGATGGA |
| rad53 | H634G_05807 | hypothetical protein H634G_05807 | CAAGAACGAGGCGGATGA |
|  |  |  | TTGACGGGAGACGAAAGC |
| top1 | E5D57_004866 | DNA topoisomerase 1 | TGGTCCAGTTGATGATGC |
|  |  |  | GAAGAGGAAGAAAGGCTATG |
